# Kaposiform Hemangioendothelioma of the GI Tract: An Exception to Occam's Principle in an Adult with SBO

**DOI:** 10.1155/2019/3269326

**Published:** 2019-04-04

**Authors:** Luis E. Aguirre, Robert A. Ali, Darcy A. Kerr, Mahsa Khanlari, Gilberto Lopes

**Affiliations:** ^1^Department of Internal Medicine, University of Miami Miller School of Medicine/Jackson Memorial Hospital, USA; ^2^Sylvester Comprehensive Cancer Center, University of Miami Miller School of Medicine, USA; ^3^Department of Pathology, University of Miami Miller School of Medicine/Jackson Memorial Hospital, USA

## Abstract

Kaposiform hemangioendothelioma (KHE) is a rare and locally aggressive vascular tumor with histological features resembling Kaposi sarcoma and capillary hemangioma mainly occurring in children and adolescents. Approximately 200 cases have been reported since its original description in 1993, with the vast majority presenting at an early age as raised ill-defined lesions with a red-blue hue mainly involving the skin and soft tissues in the extremities. Cases in adults remain extremely rare. Herein, we describe the case of a 29 year-old man who presented with progressive abdominal pain for 4 months and signs of obstipation found to be consistent with small bowel volvulus. The patient underwent exploratory laparotomy and resection of 55 cm of necrotic small bowel followed by enteroenterostomy and anastomosis. Microscopic examination revealed KHE involving small intestinal mesentery, muscularis propria, and submucosa. His recovery was uneventful and he was discharged after stabilization, opting to manage him expectantly with abdominopelvic imaging and to monitor for development of Kasabach-Merritt phenomenon. To our knowledge, this represents the first reported case of this entity presenting as intestinal obstruction in an adult for which we also present a review of the existing literature and possible treatment options.

## 1. Introduction

Kaposiform hemangioendothelioma (KHE) is a rare, locally aggressive vascular neoplasm with histological features resembling Kaposi sarcoma and capillary hemangioma that mainly occurs in children and adolescents. Reports in adults are exceedingly rare. An extensive search in PubMed using the keywords “Kaposiform” and “hemangioendothelioma” failed to reveal any reports of KHE presenting as intestinal obstruction outside of early childhood. To the extent of our knowledge, this represents the first reported case of this entity presenting as intestinal obstruction in an adult, an unusual presentation in an atypical patient population.

## 2. Case Presentation

A previously healthy 29-year-old obese man of Hispanic descent with no significant past medical or surgical history presented with complaints of progressive epigastric and periumbilical abdominal pain of 4-month duration, with an acute exacerbation 2 days prior to his initial visit in our institution. He also experienced associated nausea and emesis, fevers, and chills with obstipation and no passage of flatus. On admission, he was tachycardic and febrile. On physical examination, he had a distended abdomen, which was also diffusely tender to palpation. There were audible borborygmi. History and physical exam were concerning for small bowel obstruction.

Routine laboratory investigations were unremarkable, except for mild hyponatremia and hypochloremia. A computerized tomography (CT) scan of the abdomen and pelvis with contrast revealed multiple dilated loops of small bowel in the midline upper abdomen with thickening of the intestinal wall, mucosal hyperenhancement, and fecalization of small bowel loops which appeared to loop on themselves, suggesting small bowel volvulus. Imaging further revealed adjacent inflammatory changes in the mesentery characterized as fat stranding, multiple enlarged mesenteric lymph nodes, questionable pneumatosis intestinalis, and free fluid in the pelvis with no evidence of free air.

The patient underwent emergent exploratory laparotomy and subsequent resection of 55 cm of grossly necrotic small bowel followed by primary enteroenterostomy and end-to-end anastomosis. Copious volume of hemorrhagic fluid was present within the abdomen prior to evisceration of the small bowel.

**Figure 1:**
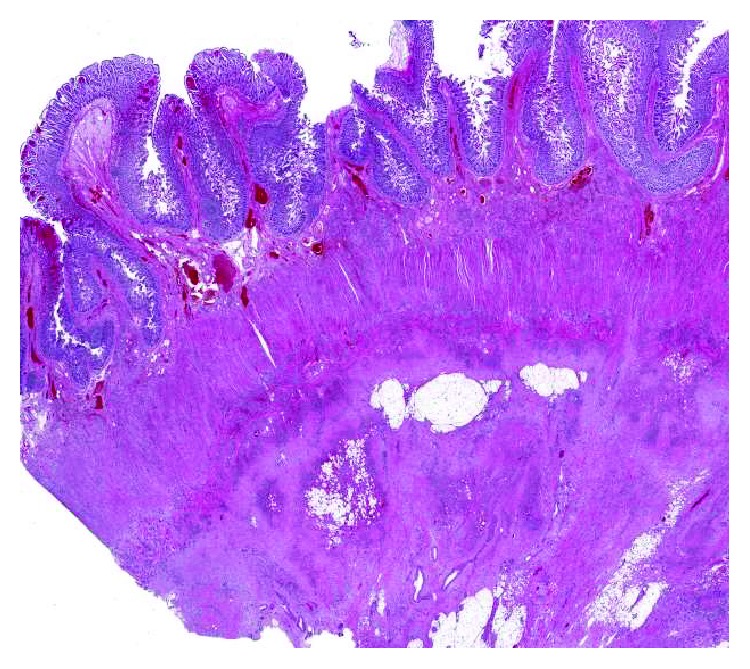
Kaposiform hemangioendothelioma histology. A scanning low-power view shows a lobulated to infiltrative vascular tumor involving small bowel mesentery, serosa, muscularis propria, and submucosa. Tumor is visible as areas of dark blue to purple vascular tissue associated with lighter pink regions of fibrosis.

**Figure 2:**
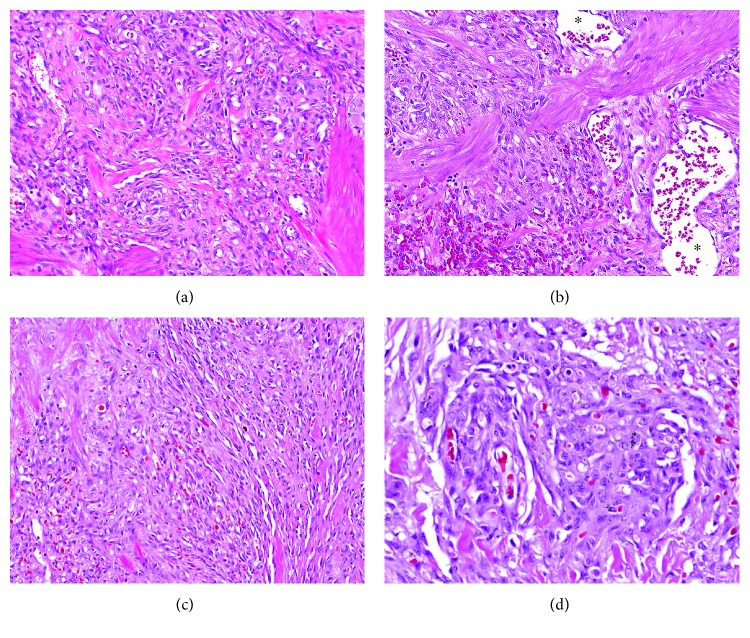
Kaposiform hemangioendothelioma histology. (a) The tumor demonstrates clusters of small capillary-sized vessels containing red blood cells, similar to capillary hemangioma. (b) Adjacent to these vascular nodules are scattered ectatic, irregularly shaped vascular channels (asterisks) with a lymphangioma-like morphology. (c) Interspersed throughout the tumor are more cellular regions composed of spindle cells reminiscent of Kaposi sarcoma but without significant cytologic atypia, mitotic activity, or necrosis. (d) The tumor is punctuated by glomeruloid structures, present as rounded nodules of dense vascular tissue associated with red blood cell fragments, hyaline droplets, and finely granular hemosiderin.

**Figure 3:**
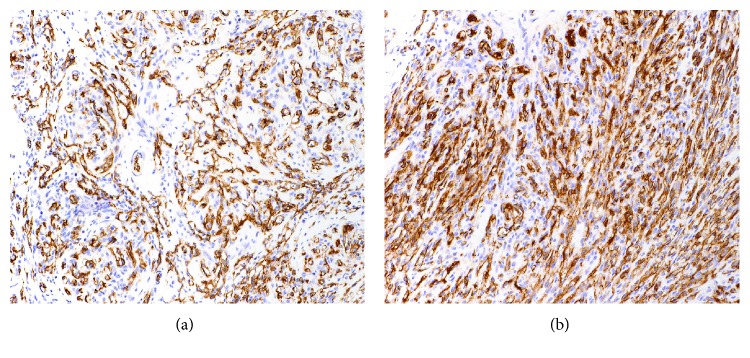
Kaposiform hemangioendothelioma immunohistochemistry. An immunohistochemical stain for D2-40 highlights the endothelial tumor cells throughout the lesion, including in the capillary hemangioma-like areas (a) as well as the more cellular Kaposi sarcoma-like regions (b).

Gross examination of the small bowel showed brown, dusky, and focally granular serosa. The mesentery was markedly firm, fibrotic, and focally retracted the intestinal wall. No clear perforations or fistula were identified. Upon opening, the mucosa was brown and edematous. Serial sections through the specimen revealed hemorrhagic and fibrotic cut surfaces without the presence of a distinct mass. Microscopic examination showed a lobulated to infiltrative vascular neoplasm involving adipose tissue, serosa, muscularis propria, and submucosa (Figure 1). The neoplasm was characterized by variable morphology, composed of nodules of small capillaries containing red blood cells and resembling capillary hemangioma (Figure 2(a)) interspersed with ectatic, irregularly shaped vascular channels resembling lymphangioma (Figure 2(b)). Interspersed throughout the tumor were cellular regions composed of loose fascicles of spindle cells associated with extravasated red blood cells and slit-like vascular spaces, reminiscent of Kaposi sarcoma (Figure 2(c)). The spindle cells had oval nuclei with vesicular chromatin and demonstrated no significant cytologic atypia, mitotic activity, or necrosis. Punctuated throughout the tumor were scattered glomeruloid structures, present as rounded nodules of vessels associated with red blood cell fragments, hyaline droplets, and finely granular hemosiderin deposition (Figure 2(d)), characteristic of KHE. Immunohistochemical stains revealed that the tumor cells were strongly positive for the vascular endothelial markers CD34 and CD31. D2-40 (podoplanin), a lymphatic endothelial marker, highlighted cells in both capillary hemangioma-like areas and spindle cells areas (Figure 3). A stain for HHV8 was negative, helping to exclude Kaposi sarcoma. The constellation of morphologic and immunohistochemical features was diagnostic of KHE involving small intestinal mesentery, muscularis propria, and submucosa.

Clinically, the patient had normal platelet levels, excluding an association with consumptive coagulopathy leading to thrombocytopenia (Kasabach-Merritt phenomenon). No complications were reported during the procedure or in the immediate "post-operative period", and the patient was discharged after stabilization and recovery of intestinal function with outpatient follow-up planned shortly thereafter. At the time of this report, the patient is stable, with no evidence of disease 5 months after surgery. We opted to manage him expectantly with serial CT scans of the abdomen and pelvis and to monitor for signs and symptoms that would suggest the development of Kasabach-Merritt phenomenon.

## 3. Discussion

Kaposiform hemangioendothelioma (KHE) is a rare, locally aggressive vascular neoplasm with histological features resembling those of Kaposi sarcoma (spindle-shaped endothelial cells and slit-like vascular channels) and capillary hemangioma mainly presenting in children and adolescents [1]. More than 50% of cases are diagnosed within the first year of life [2]. The entity was first described by Zukerberg et al. in 1993, when it was found to be associated with thrombocytopenia and consumption coagulopathy (Kasabach-Merritt phenomenon) [3]. To date, approximately 200 cases have been reported in the literature with the vast majority of them presenting at an early age (childhood and infancy period) as raised ill-defined lesions with a red-blue hue involving the skin and soft tissues in the extremities, followed by the retroperitoneum (most common extracutaneous location), muscle, bone, thoracic cavity, mediastinum, lymph nodes, head, and neck as well as intra-abdominal organs [1, 4]. Cases in adults remain extremely rare with 2 reports of lesions involving the testes and 2 cases involving the thoracic cavity or cage [5–7].

KHE is not associated with HHV8 infection like Kaposi sarcoma; its etiology is unknown [2]. Immunohistochemically, the spindle cells are positive for vascular endothelial markers (CD31, CD34, and ERG) but not for GLUT1 (which is positive in the endothelial cells of infantile hemangioma) [3]. Smooth muscle actin (SMA) is focally positive within the tumor mass indicating the presence of pericytes. The slit-like lymphangiomatous areas exhibiting rich lymphatic vessel configuration show positive staining for D2-40 (podoplanin).

KHE is a locally aggressive neoplasm. Exceptionally rare reports of metastasis are present in literature [2]. However, approximately 10% of patients die as a consequence of disease, either due to local growth or Kasabach-Merritt phenomenon [2]. Up to 70% of patients with KHE develop Kasabach-Merritt phenomenon, the risk of which seems to be highest with large lesions and congenital lesions and with tumors located in the mediastinum and retroperitoneum [8].

As for the management of patients with KHE, the most critical points to discern are if there is an association with Kasabach-Merritt phenomenon to determine the need for hemostasis and whether the lesion is sizeable and symptomatic enough to warrant treatment. Strategies range from complete surgical excision (which may be difficult given that tumor margins are often poorly defined) to laser therapy and chemotherapy (involving the use of agents either alone or in combination) if tumor is not amenable to resection [9]. The latter scenario usually involves the use of prednisone with adjunctive aspirin as first-line treatments before opting for chemotherapy regimens involving vincristine, propranolol, sirolimus, or interferon alpha. Other options also include radiation and embolization [10].

In summary, Kaposiform hemangioendothelioma represents an exceedingly rare entity usually affecting children and adolescents with only 4 cases reported in adults in the literature. Our case of a 29-year-old man presenting with intestinal obstruction is unusual with regard to age as well as location and pattern of presentation. An extensive literature search reveals a single instance of KHE presenting as intestinal obstruction involving a sixteen-month-old boy [11], but no cases of this presentation in adults had been described thus far. Awareness of unusual presentations of KHE such as in this case illustrates how timely surgical intervention and a proper histopathological diagnosis may prevent potentially catastrophic consequences, namely, thrombocytopenia and severe bleeding diathesis requiring urgent hemostasis.
